# Role of the larval feeding morphology and digestive enzyme activity in the early development of the polychaete *Boccardia wellingtonensis*

**DOI:** 10.7717/peerj.6151

**Published:** 2019-01-04

**Authors:** Daniel Doherty-Weason, Fernanda X. Oyarzun, Luciano Vera, Miguel Bascur, Fabián Guzmán, Francisco Silva, Ángel Urzúa, Antonio Brante

**Affiliations:** 1 Facultad de Ciencias, Universidad Católica de la Santísima Concepción, Concepción, Chile; 2 Centro de Investigación en Biodiversidad y Ambientes Sustentables (CIBAS), Universidad Católica de la Santísima Concepción, Concepción, Chile; 3 Centro i∼mar, Universidad de Los Lagos, Puerto Montt, Chile

**Keywords:** Life history strategy, Fluorescence microscopy, Larval feeding behavior, Marine invertebrates, Poecilogony, Adelphophagy

## Abstract

In marine invertebrates, the modes of development at early stages are related to the type and capacity of larval feeding to achieve growth. Therefore, studying the factors that determine larval feeding strategies can help to understand the diversity of life histories and evolution of marine invertebrates. The polychaete *Boccardia wellingtonensis* is a poecilogonous species that encapsulates and incubates its offspring. This species produces two types of larvae: (1) larvae that do not feed within the capsule and hatch as planktotrophic larvae (indirect development), and (2) adelphophagic larvae that feed on nurse eggs and other larvae inside the capsule to hatch as advanced larvae or juveniles (direct development). Otherwise, the larval types are indistinguishable at the same stage of development. The non-apparent morphological differences between both types of larvae suggest that other factors are influencing their feeding behavior. This work studied the potential role of the activity of 19 digestive enzymes on the different feeding capacities of planktotrophic and adelphophagic larvae of *B. wellingtonensis*. Also, differences in larval feeding structures and the larval capacity to feed from intracapsular fluid were evaluated by electron and fluorescence microscopy. Results showed that both types of larvae present similar feeding structures and had the capacity to ingest intracapsular fluid protein. Adelphophagic larvae showed overall the highest activities of digestive enzymes. Significant differences between larval types were observed in nine enzymes related to the use of internal and external nutritional sources. Given that larval feeding is closely related to larval development in species with encapsulation, this work supports that the study of the digestive enzymatic machinery of larvae may contribute to understanding the evolution of developmental modes.

## Introduction

In the context of species with complex life cycles, the study of factors that determine the characteristics of early life stages is relevant to understanding the evolution of developmental modes. Marine invertebrates present a great diversity of patterns of larval development, which have been generally categorized by the dichotomy between a free-swimming larval stage (i.e., indirect developmental mode) and the absence of such stage (i.e., direct developmental mode) (Thorson, 1950; Mileikovsky, 1971; Krug et al., 2015). Several ecological and evolutionary aspects such as population connectivity, speciation rate and extinction probability are influenced by developmental mode (Jablonski & Lutz, 1983; Krug et al., 2015). In species with encapsulation, the development of larvae depends on the availability of food provided by the female within capsules and on their capacity to use it; therefore, larval developmental modes are related to the capacity to acquire and assimilate food. When intracapsular food is present, larvae with high feeding capacity are able to feed and grow to advanced stages of development within the capsule (as in direct development), while larvae with low intracapsular food and low feeding capacity hatch earlier to complete development in the plankton (as in indirect development) (Thorson, 1950; Vance, 1973 Collin, 2003). Thus, the study of factors determining different larval feeding strategies in marine invertebrate species may shed light on the evolution of developmental modes in the marine realm.

Factors that determine larval feeding capacity and nutritional sources have been used as central aspects to understand the evolutionary transitions and reversions between feeding strategies and developmental modes in marine invertebrate larvae (Strathmann, 1978; Jablonski & Lutz, 1983; Collin, 2004; Krug et al., 2015). Based on the nutritional sources of larvae, different feeding strategies have been defined: (1) planktotrophy, in which free swimming larvae develop and feed in the plankton from an external source of food; (2) lecitotrophy, free living or encapsulated larvae that feed from a parentally derived internal yolk reserve; (3) ovophagy, encapsulated larvae that feed on nurse eggs and develop in the capsule; and (4) adelphophagy, encapsulated larvae that develop by feeding on other larvae and embryos within the capsule (Thorson, 1950; Strathmann, 1978; Fenchel & Christiansen, 1979; Collin & Spangler, 2012). Each feeding strategy is associated with the presence or absence of specific morphological structures of larvae that allow feeding. For example, the ciliated velum of planktotrophic veliger larvae of marine gastropods allow them to swim and capture food after hatching (Thompson, 1959; Moran, 1999; Fretter & Montgomery, 1968; Cumplido et al., 2011). In contrast, adelphophagic and ovophagic larvae of calyptraeid gastropods have a reduced velum, given that ingestion occurs by engulfing or by degradation of nurse eggs or other embryos with cilia (Chaparro, Charpentier & Collin, 2002; Véliz, Winkler & Guisado, 2003; Brante, Fernández & Viard, 2013). In this manner, larval developmental mode is determined by the morphologically derived capacity of larvae to use the nutritional source available inside capsules, allowing them to grow to advanced stages of development when food is available.

In spite of the importance of feeding structures determining larval development, there are some species of polychaetes and gastropods in which larvae have different feeding strategies in spite of no apparent difference in anatomical structure associated with food intake (Krug, Gordon & Romero, 2012; Gibson & Carver, 2013; McDonald, Collin & Lesoway, 2014; Oyarzun & Brante, 2014; Oyarzun & Brante, 2015). The fact that larvae with different feeding strategies and different modes of development have similar feeding morphology suggests that an additional mechanism, other than feeding structures and food ingestion capability, may be influencing larval feeding types, and therefore their mode of development. The role of digestive enzymes in the development of marine invertebrate larvae has been extensively reviewed in crustaceans (Jones et al., 1997; Rotllant et al., 2008), and scarcely in gastropods (Collin & Starr, 2013). Research on the enzymatic digestive machinery in larvae presents a challenging opportunity to understand larval feeding types and their evolution. For example, in the calyptraeid group, Collin & Starr (2013) showed that digestive enzyme activity increases during the ontogeny of larvae with different feeding strategies, and that the type and activity of specific digestive enzymes is different between planktotrophic, adelphophagic and non-feeding larvae. Planktotrophic larvae showed the highest digestive enzyme activity, followed by adelphophagic larvae and finally by lecithotrophic larvae with large yolk reserves (Collin & Starr, 2013).

Differences in digestive enzyme activity may be related to the nutritional requirements of each of the modes of development, since a high specific enzyme activity in the early stages of development would provide larvae with a high capacity to feed on specific types of food, allowing them to advance in development. In the study of Thomsen, Collin & Carrillo-Baltodano (2014), adelphophagic and planktotrophic larvae of calyptraeid gastropods fed with sibling tissue and yolk showed no differences in allometry; however, adelphophagic larvae had a higher development rate and consumed more tissue and yolk than planktotrophic larvae (Thomsen, Collin & Carrillo-Baltodano, 2014). Similar results were observed in planktotrophic and adelphophagic larvae of the polychaete *Boccardia proboscidea* (Oyarzun & Brante, 2014), in which both types of larvae share similar feeding structures during their development within the same capsule. In this species, planktotrophic larvae did not feed or advance in development when presented with whole or mashed nurse eggs, while adelphophagic larvae fed on the nurse eggs and developed; however, when presented with phytoplankton, all larvae were able to consume it and grow and their ontogenic tragectory was indistinguisable among them (Oyarzun & Brante, 2014). This shows that in spite of sharing similar ingestion capacity, adelphophagic larvae have a higher capacity to feed and develop from a tissue and yolk diet than planktotrophic larvae, which may be related to the presence and activity of specific digestive enzymes associated with the digestion of these nutritional sources.

**Figure 1:**
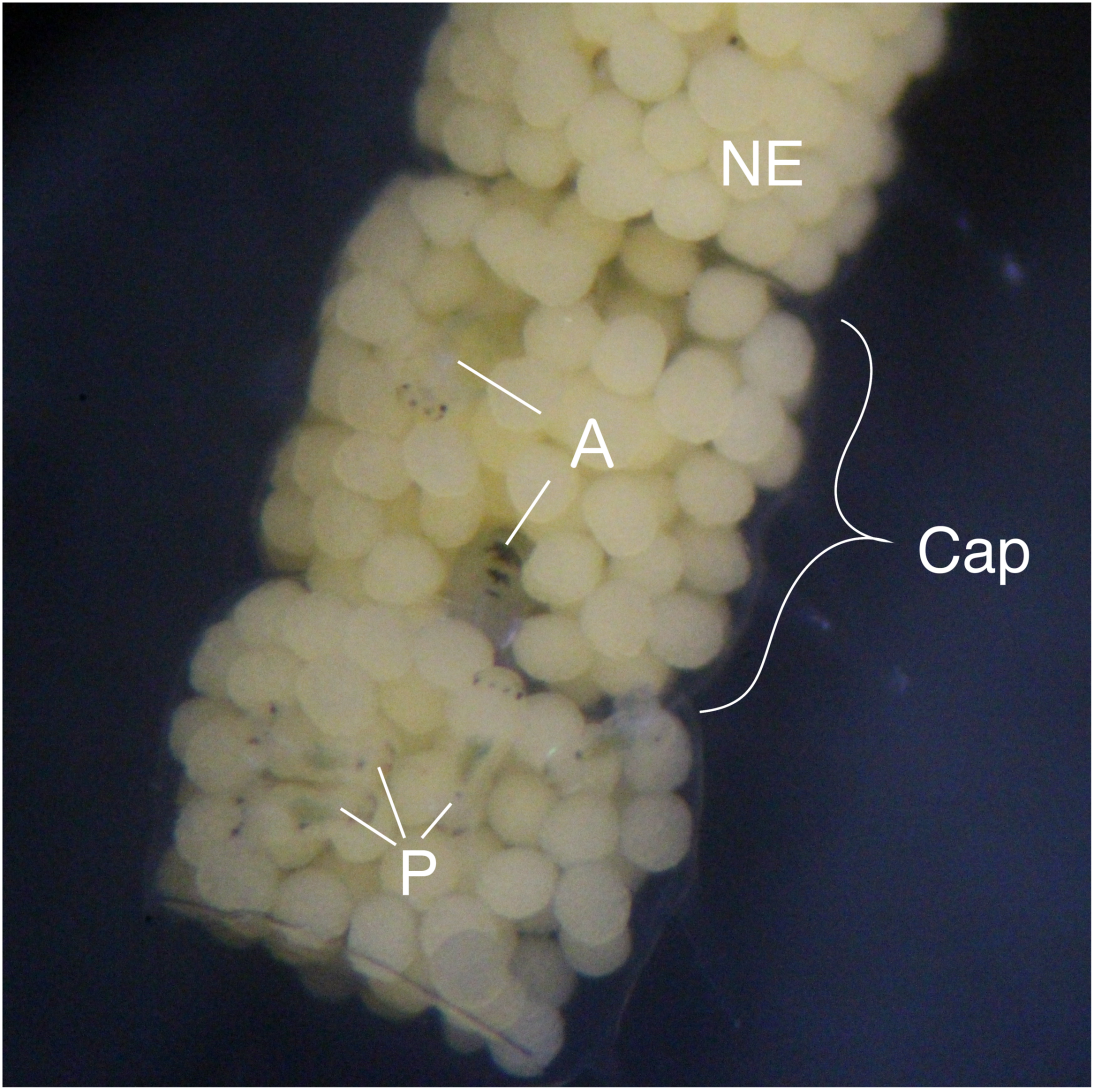
Type III egg mass of *B. wellingtonensis*. Egg mass with capsules (Cap) containing nurse eggs (NE), planktotrophic larvae (P), and adelphophagic larvae (A).

*Boccardia wellingtonensis* (Read, 1975) is a spionid polychaete that encapsulates and broods its offspring, and inhabits the intertidal zone of New Zealand, South Africa and Chile (Simon et al., 2010; Oyarzun & Brante, 2015). This species shows poecilogony, which consists of the production of different types of larvae represented in two modes of reproduction: Type I: females produce only planktotrophic larvae that do not feed within the capsule and hatch at early stage; Type III: females produce planktotrophic larvae and adelphophagic larvae that feed on the nurse eggs and other larvae (Oyarzun & Brante, 2015) (Fig. 1). In the early stages, both planktotrophic and adelphophagic larvae have similar morphology and are indistinguishable; however, shortly after the first segment starts appearing, some larvae begin to feed on nurse eggs and other embryos within the capsule (adelphophagy) growing to advanced stages of development and hatching as juveniles. On the other hand, another group of larvae do not feed and stays at earlier stages of development until hatching occurs, and then continue their development in the plankton as planktotrophic larva. Having both types of development within a single species presents a good model to evaluate the evolution of larval feeding types and developmental modes, given the lack of phylogenetic effects which may obscure underlying patterns (Blomberg & Garland, 2002; Collin, 2004). In this work, the pattern of the digestive enzymatic activity of larvae of the poecilogonous polychaete *B. wellingtonensis* with different feeding strategies (planktotrophy and adelphophagy) was studied. We tested the hypothesis that adelphophagic and planktotrophic larvae show different digestive enzyme profiles, with a higher activity of specific digestive enzymes associated with the digestion of nurse eggs (lipids and protein) in adelphophagic larvae in comparison with planktotrophic larvae.

## Materials and Methods

### Larval collection and rearing

**Figure 2:**
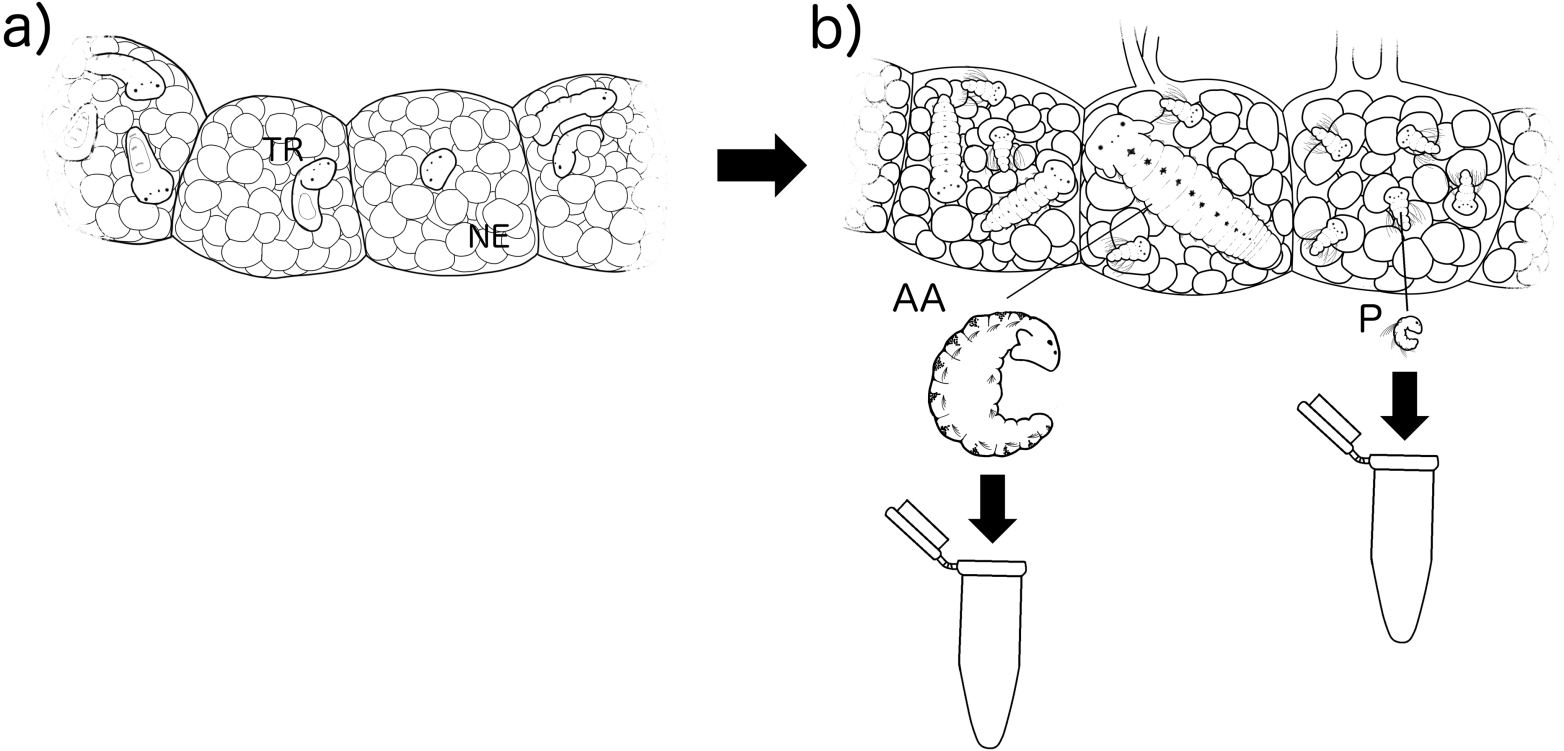
Experimental methodology of larval rearing. Larvae were reared inside their capsules with nurse eggs (NE) from their (A) early trochophore stage (TR) (point in time in which their feeding type cannot be identified), to (B) when they can be identified by their size in number of setigers as alphophagic (AA) with 10–12 setigers and planktotrophic (P) with 3–5 setigers.

*Boccardia wellingtonensis* capsules from Type I and Type III females were collected from the intertidal zone of Coliumo, Biobío region, Chile (36°32′57.23″S. 72°57′18.52″W) between August and December 2017. Additionally, in order to compare digestive enzymes between larvae of poecilogonous and non-poecilogonous polychaete species, capsules of *B. chilensis*, a closely related species that produces only planktotrophic larvae, were collected from the locality of Lirquén, Biobío region, Chile (36°42′3.41″S. 72° 58′32.32″W) during August and December 2017. Following the methodology of Oyarzun & Brante (2015) and Strathmann (1987), each capsule was reared inside a 1.5 ml eppendorf tube with 1 mL of 40 µm mesh filtered and sterilized seawater with antibiotics (50 µg/mL of penicillin and 50 µg/mL of streptomycin) at constant temperature (20 °C) and 18:6 light:dark photoperiod. Water was changed each second day to ensure quality and avoid oxygen depletion. Capsules were reared until larvae were developed enough to identify the feeding strategy of each larva according to Oyarzun & Brante (2015). The developmental stage of the larvae was measured by the number of segments with chaeta (setigers) as it is usually done in polychaetes. Then, larvae were collected and pooled depending on their type: (1) Type I planktotrophic larvae: only planktotrophic larvae are observed in a brood (3–5 setigers in size); (2) Type III planktotrophic larvae: planktotrophic larvae (3–5 setigers in size) occurring with adelphophagic larvae and nurse eggs in a brood; (3) adelphophagic larvae (10–12 setigers) (Fig. 2). For *B. chilensis* only planktotrophic larvae were used.

### Scanning electron microscopy

In order to evaluate potential morphological differences in the external feeding structures of *B. wellingtonensis* larvae that may influence feeding capacity, electron microscopy photographs were taken from early stage adelphophagic, Type I planktotrophic and Type III planktotrophic larvae, which were fixed and observed with an electron microscope with the methodology of Gibson & Carver (2013).

### Fluorescence microscopy photography

One of the main components of nurse eggs and intracapsular fluid of species with encapsulation is protein (Chaparro, Charpentier & Collin, 2002; Brante, Fernandez & Viard, 2009; Chaparro et al., 2012). Then, to evaluate the capacity of different larval types to ingest intracapsular dissolved protein, fluorescently marked albumin (protein) (FITC-BSA, Sigma A9771) was used following the methodology by Brante, Fernandez & Viard (2009). Adelphophagic, Type III planktotrophic and Type I planktotrophic larvae of *B.  wellingtonensis* were placed in a solution of seawater and fluorescent albumin at concentrations of 0.5 and 1 mg/L for periods of 2, 4, and 6 h. Then, larvae were rinsed with sterile seawater and fixed before being observed under an epifluorescence microscope with a FITC filter set. Given that some organisms have their own fluorescence, which in this case may interfere with the analysis, a set of control of larvae were placed in plain seawater under the same time periods and observed under the epifluorescence microscope. Due to their small size, more exposure time was necessary for planktotrophic larvae in order to observe the fluorescence.

### Digestive enzymatic analysis

The digestive enzymatic activity of larvae of *B. wellingtonensis* of different feeding types (described above) and for planktotrophic larvae of *B. chilensis* was evaluated. A total of 300 planktotrophic larvae (0.6 µg each) and 13 adelphophagic larvae (13.7 µg each) was necessary to obtain about 180 ug of each dry weight sample, the minimum amount to run the enzymatic test. Given the high number of larvae needed for analyses and the low frequency of Type III planktotrophic larvae in broods, only three replicates were carried out for different larval types of *B. wellingtonensis* and *B. chilensis*. The enzymatic analysis was done with the Api-Zym REF 25–200 kit (bioMériux, France) following the methodology of Collin & Starr (2013). A total of 19 enzymes of four major groups were measured: peptide hydrolases (leucine arylamidase, valine arylamidase, cystine arylamidase, trypsin, and α chymotrypsin); ester hydrolases (esterase (C4), esterase lipase (C8), and lipase (C14)); phosphoric hydrolases (alkaline phosphatase, acid phosphatase, and naphthol-AS-BI phosphohydrolase); and glycosidases (α galactosidase, β galactosidase, β glucuronidase, α glucosidase, β glucosidase, N-acetyl-, β glucosaminidase, α mannosidase, and α fucosidase). Before the analyses, each sample was thawed, placed in distilled water and homogenized. Then, each well of one test strip was inoculated with 75 µl of a single homogenized sample and incubated at 37 °C for 4.5 h. After incubation, one drop of the Api-Zym A and one of Api-Zym B reagents were added to each well to stop the reaction and were set to rest for 10 min. To quantify enzymatic activity, 90 µL of each sample were added to a microplate to measure absorbance at 450 nm in a spectrophotometer (Biotek Elx808).

### Statistical analysis

Digestive enzymatic activity was compared between larval feeding strategies with one-way ANOVAs for each of the specific enzymes, and Tukey *a posteriori* tests were run when significant differences were observed. Previous to statistical analyses, the normality of the data was tested with Shapiro-Whilk test and homogeneity with the Cochrane test. Finally, a Principal Component Analysis (PCA) was performed to compare digestive enzymatic profiles between different larval types. Statistical analyses were run in the software Statistica 7 for ANOVAs, and PRIMER 6 (Plymouth Routines in Multivariate Ecological Research) (Clarke & Gorley, 2015) for multivariate analyses.

## Results

### Electron microscopy photography

**Figure 3:**
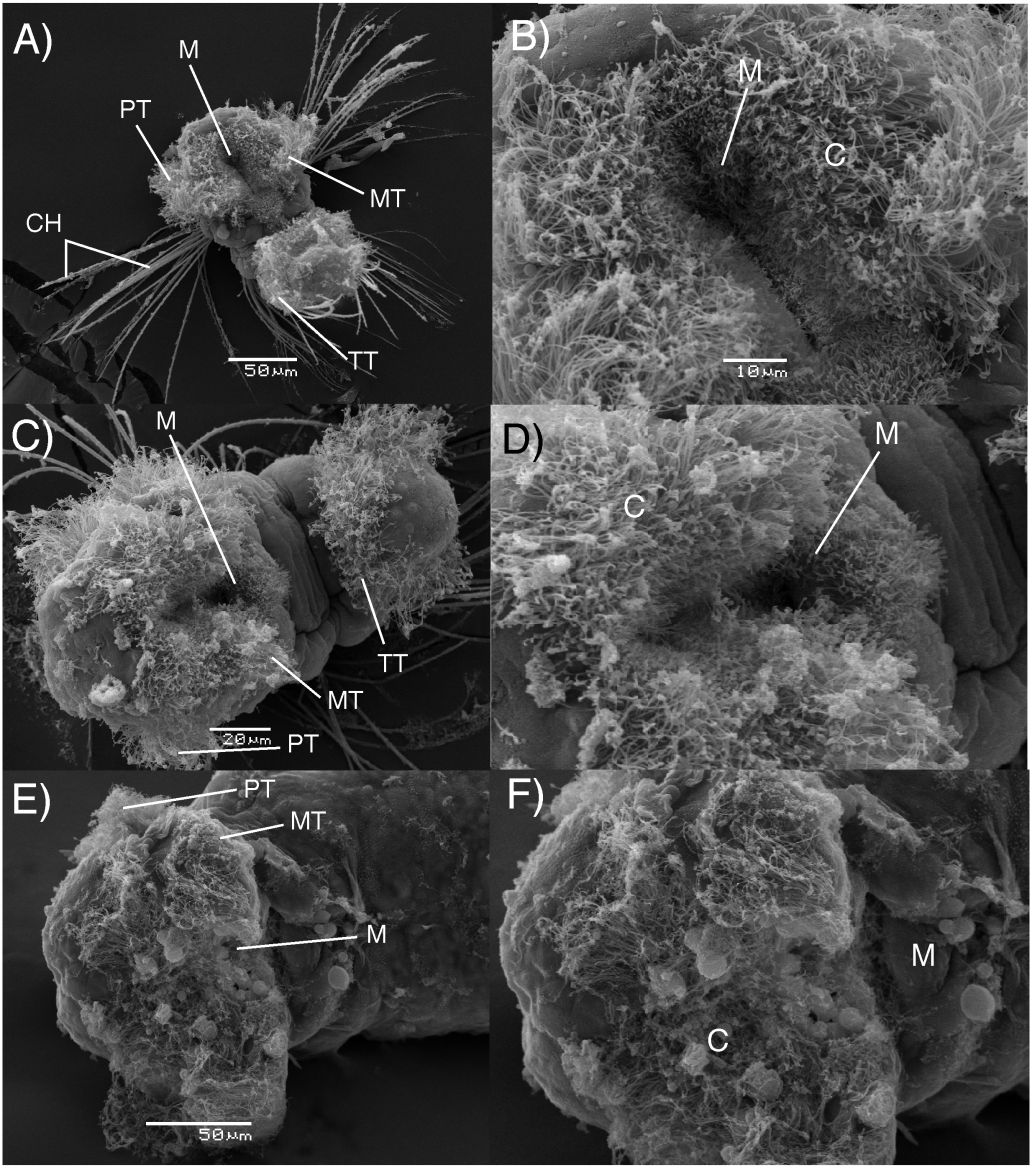
Electron microscopy images of *B. wellingtonensis* larvae. Type I planktotrophic larva (A), Type III planktotrophic (B), adelphophagic larva (C) and magnification of mouth of each larva (B, D, F). Swimming trochs present: prototroch (PT) metatroch (MT) and telotroch (TT), together with chaetae and feeding structures: M, mouth; C, cilia.

The results of electron microscopy showed that there were no apparent differences in the feeding structures between adelphophagic, Type I and Type III planktotrophic larvae of *B. wellingtonensis* (Fig. 3). Mouths and feeding cilia were observed in all three kind of larvae, together with the presence of the swimming trochs such as prototroch, metatroch and telotroch, although, elongated chaeta were present only in planktotrophic larvae (Fig. 3).

### Fluorescence microscopy photography

**Figure 4:**
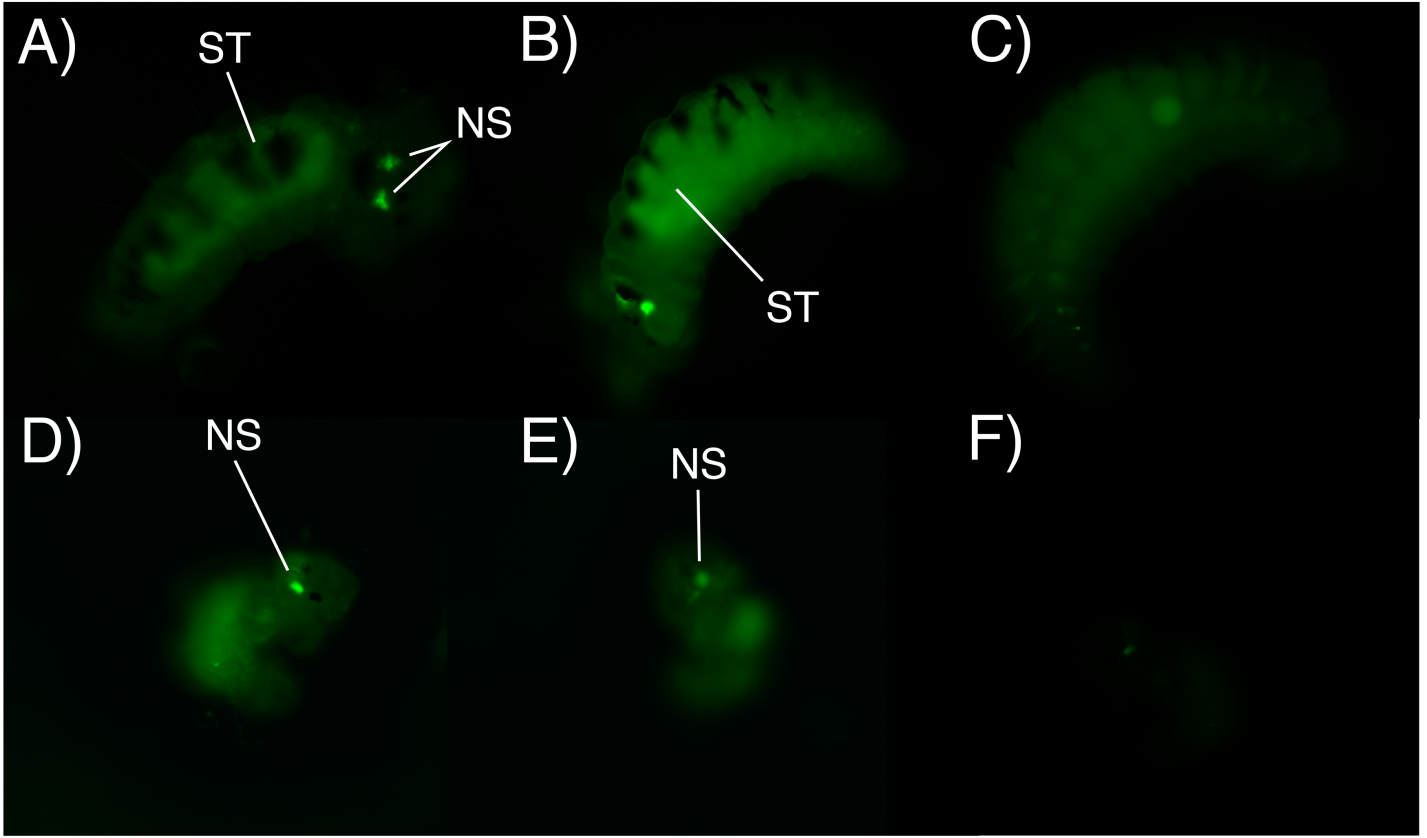
Fluorescence microscopy photographs of *B. wellingtonensis* larvae. Fluorescence microscopy photographs of *B. wellingtonensis* larvae (A) adelphophagic larva in 0.5 mg/L of fluorescent albumin and 3 s exposure photography, (B) adelphophagic larva in 1mg/L of fluorescent albumin and 3 s exposure photography, (C) adelphophagic larva without fluorescent albumin and 3 s exposure photography (control), (D) Type I planktotrophic larva in 0.5 mg/L of fluorescent albumin and 10 s exposure photography, (E) Type III planktotrophic in 0.5 mg/L of fluorescent albumin and 10 s exposure photography, and (F) Type III planktotrophic larva in 0.5 mg/L of fluorescent albumin and 3 s exposure photography. NS (nuchal structure), GT (gut).

After 6 h of incubation in the fluorescent solution, some larval structures became visible with fluorescence at both concentrations (0.5 mg/L and 1 mg/L), with the strongest signal observed at the higher concentration of the marked albumin. Fluorescence was observed in the digestive tract and stomach of both adelphophagic and planktotrophic larvae (Fig. 4). Additionally, two fluorescent spots were observed on the top anterior end of both larval types, which should correspond to the nuchal organ (Fig. 4), which generally consist of a group of cilia that mainly function as a sensory organ (Purschke, 1997).

### Digestive enzyme analysis

**Figure 5:**
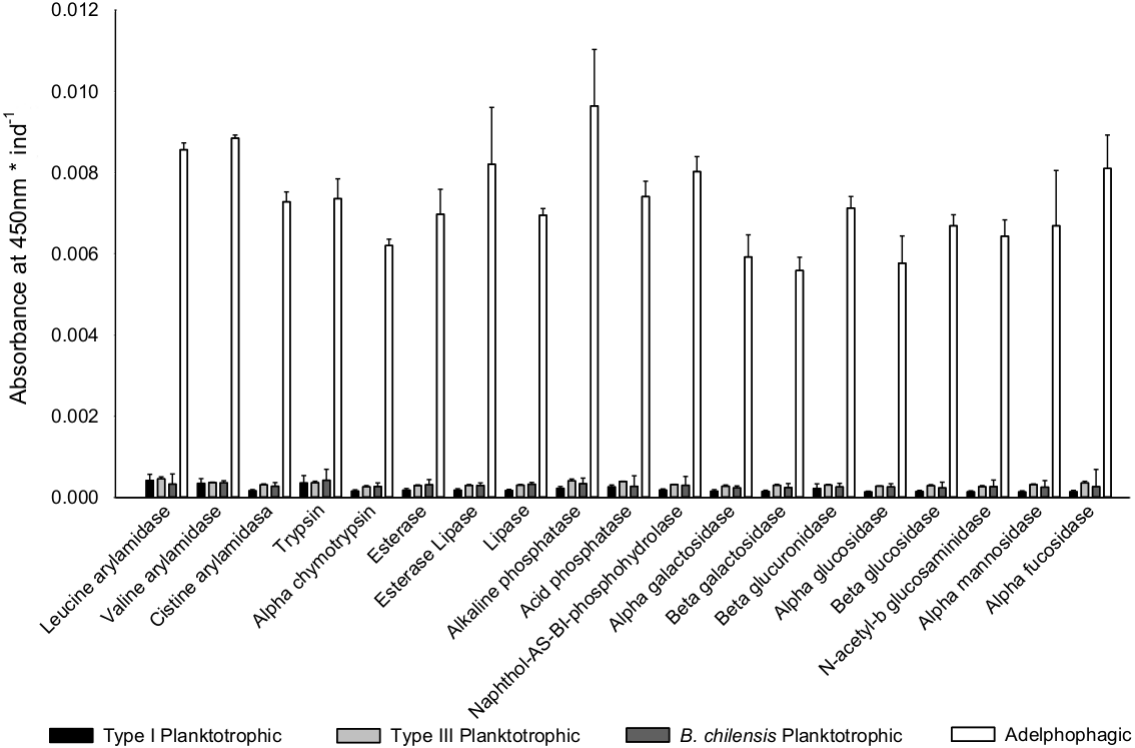
Digestive enzyme activity of polychaete larvae. Enzymatic activity is expressed as absorbance per individual larva of planktotrophic Type I (PTI), planktotrophic Type III (PTIII) and adelphophagic (ADF) larvae of *B.  wellingtonensis*, and planktotrophic larvae of *B. chilensis* (PBC).

Once differences in larval growth and size allowed to recognize different larval types, the general pattern of the enzymatic digestive activity showed that the highest absorbance per larva was observed in adelphophagic larvae in comparison to other feeding strategies, including larvae of *B. chilensis* (Fig. 5). However, when enzymatic activity was standardized by the total amount of larval tissue, enzymatic profiles showed that difference between Type III planktotrophs and adephophages is less striking than the difference between larvae in Type III reproduction and reproduction solely with planktotrophs (planktotrophic larvae Type I of *B. wellingtonensis* and planktotrophic larvae of *B. chilensis*). More specifically, the one-way ANOVAs detected significant differences between larval types in nine out of 19 enzymes: three peptide hydrolases: leucine arylamidase, cystine arylamidase and trypsin; two phosphoric hydrolase acids: acid phosphatase and naphthol-AS-BI phosphohydrolase; and four glycosidases: *β* galactosidase, *β* glucuronidase, *β* glucosidase and *α* fucosidase (Table 1). However, the Tukey *a posteriori* tests showed no significant differences between any of the larval types for enzymes cystine arylamidase and *β*-glucosidase. In the rest of enzymes, the general pattern showed that the enzymatic activity of adelphophagic larvae was higher and differed significantly from Type I planktotrophic larvae of *B. wellingtonensis* and planktotrophic larvae of *B. chilensis* (Table 2). Levels of the enzymatic activity of planktotrophic larvae Type III were more similar to adelphophagic larvae (Table 2).

**Table 1:** Comparison of the digestive enzymatic activity between larval types of *Boccardia wellingtonensis* and *B. chilensis*. Results of the one-way ANOVAs comparing the enzymatic activity (standardized to 180 mg of larval tissue dry weight) among different types of larvae of *B. wellingtonensis* (planktotrophic Type I, planktotrophic Type III and adelphophagic larvae) and planktotrophic larvae of *B. chilensis*.

Enzyme	Factor	*df*	MS	*F*	*P*
Alkaline phosphatase	Larvae	3	4.94E–04	0.99	0.44^ns^
	Error	8	4.99E–04		
	Total	11	9.93E–04		
Esterase	Larvae	3	2.70E–04	1.83	0.22^ns^
	Error	8	1.48E–04		
	Total	11	4.18E–04		
Esterase Lipase	Larvae	3	4.88E–04	1.68	0.25^ns^
	Error	8	2.91E–04		
	Total	11	7.80E–04		
Lipase	Larvae	3	1.30E–04	3.85	0.06^ns^
	Error	8	3.36E–05		
	Total	11	1.63E–04		
Leucine arylamidase	Larvae	3	1.11E–03	7.13	<0.05^*^
	Error	8	1.55E–04		
	Total	11	1.26E–03		
Valine arylamidase	Larvae	3	2.23E–04	3.22	0.08^ns^
	Error	8	6.91E–05		
	Total	11	2.92E–04		
Cystine arylamidase	Larvae	3	1.92E–04	4.12	<0.05^*^
	Error	8	4.67E–05		
	Total	11	2.39E–04		
Trypsine	Larvae	3	1.57E–03	4.49	<0.05^*^
	Error	8	3.49E–04		
	Total	11	1.92E–03		
α-chymotrypsine	Larvae	3	8.07E–05	1.43	0.31^ns^
	Error	8	5.66E–05		
	Total	11	1.37E–04		
Acid phosphatase	Larvae	3	7.68E–04	16.25	<0.001^***^
	Error	8	4.73E–05		
	Total	11	8.16E–04		
PHO	Larvae	3	1.32E–04	4.72	<0.05^*^
	Error	8	2.79E–05		
	Total	11	1.60E–04		
α-galactosidase	Larvae	3	1.70E–04	2.01	0.19^ns^
	Error	8	8.46E–05		
	Total	11	2.55E–04		
β-galactosidase	Larvae	3	2.73E–04	6.60	<0.05^*^
	Error	8	4.14E–05		
	Total	11	3.15E–04		
β-glucuronidase	Larvae	3	3.16E–04	14.54	<0.01^**^
	Error	8	2.17E–05		
	Total	11	3.37E–04		
α-glucosidase	Larvae	3	1.11E–04	1.03	0.43^ns^
	Error	8	1.07E–04		
	Total	11	2.18E–04		
β-glucosidase	Larvae	3	3.20E–04	5.03	<0.05^*^
	Error	8	6.36E–05		
	Total	11	3.83E–04		
NAG	Larvae	3	9.64E–05	0.94	0.47^ns^
	Error	8	1.03E–04		
	Total	11	1.99E–04		
α-mannosidase	Larvae	3	4.60E–04	1.73	0.24^ns^
	Error	8	2.65E–04		
	Total	11	7.25E–04		
α-fucosidase	Larvae	3	1.19E–03	6.70	<0.05^*^
	Error	8	1.77E–04		
	Total	11	1.36E–03		

**Notes.**

Differences in enzymatic activity among types of larvae were evaluated by one-way ANOVA; significant differences (*P < 0.05; **P < 0.01; ***P < 0.001; ns superscript: no significant differences).

**Table 2:** Digestive enzymatic activity in larvae of *Boccardia wellingtonensis* and *B. chilensis*. Enzymatic activities measured as absorbance at 450 nm in larvae of *B. wellingtonensis* of different feeding type: planktotrophic Type I (PTI), planktotrophic Type III (PTIII) and adelphophagic (ADF) larvae, planktotrophic larva of *B. chilensis* (PBC) detected by APIZIME^^®^^ enzyme test kit. Enzymes showing significant differences between larval type are highlighted in bold with the lowercase letters representing the result of the *a posteriori* Tukey tests. NAG: N-acetyl- β-glucosaminidase; PHO Naphthol-AS-BI phosphohydrolase PHO = Naphthol-AS-BI phosphohydrolase; NAG: N-acetyl- β-glucosaminidase. Values represent mean ± SD. mean ± SD.

****		**Type of larvae**				
**Enzyme**		**PTI**	**PTIII**	**PBC**	**ADF**	
*Ester hydrolases*						
Esterase		0.073 ± 0.001^**a**^	0.089 ± 0.008^**a**^	0.095 ± 0.019^**a**^	0.091 ± 0.014^**a**^	
Esterase Lipase		0.076 ± 0.001^**a**^	0.090 ± 0.010^**a**^	0.089 ± 0.010^**a**^	0.110 ± 0.03^**a**^	
Lipase		0.082 ± 0.001^**a**^	0.092 ± 0.008^**a**^	0.098 ± 0.008^**a**^	0.090 ± 0.004^**a**^	
*Peptide hydrolase*						
**Leucine arylamidase**		**0.096 ± 0.009**^**a**^	**0.138 ± 0.021**^**b**^	**0.099 ± 0.010**^**a,c**^	**0.110 ± 0.004**^**a,b,c**^	
Valine arylamidase		0.095 ± 0.007^**a**^	0.111 ± 0.005^**a**^	0.110 ± 0.014^**a**^	0.115 ± 0.002^**a**^	
Cystine arylamidase		0.078 ± 0.004^**a**^	0.093 ± 0.011^**a**^	0.084 ± 0.006^**a**^	0.095 ± 0.005^**a**^	
**Trypsine**		**0.074 ± 0.001**^**a**^	**0.110 ± 0.016**^**a,b**^	**0.130 ± 0.032**^**b**^	**0.095 ± 0.011**^**a,b**^	
α-chymotrypsine		0.070 ± 0.003^**a**^	0.079 ± 0.013^**a**^	0.081 ± 0.006^**a**^	0.081 ± 0.004^**a**^	
*Phosphoric hydrolase*						
Alkaline phosphatase		0.102 ± 0.012^**a**^	0.123 ± 0.020^**a**^	0.102 ± 0.020^**a**^	0.126 ± 0.030^**a**^	
**Acid phosphatase**		**0.090 ± 0.005**^**a**^	**0.120 ± 0.002**^**b**^	**0.083 ± 0.010**^**a**^	**0.096 ± 0.010**^**a**^	
**PHO**		**0.090 ± 0.004**^**a**^	**0.096 ± 0.002**^**a,b**^	**0.091 ± 0.005**^**a,b**^	**0.104 ± 0.008**^**b**^	
*Glycosidases*						
α-galactosidase		0.067 ± 0.003^**a**^	0.084 ± 0.013^**a**^	0.071 ± 0.004^**a**^	0.077 ± 0.012^**a**^	
**β-galactosidase**		**0.069 ± 0.001**^**a**^	**0.091 ± 0.010**^**b**^	**0.074 ± 0.005**^**a,b,c**^	**0.073 ± 0.007**^**a,c**^	
**β-glucuronidase**		**0.073 ± 0.002**^**a**^	**0.093 ± 0.007**^**b**^	**0.078 ± 0.001**^**a,c**^	**0.093 ± 0.006**^**b**^	
α-glucosidase		0.070 ± 0.003^**a**^	0.085 ± 0.002^**a**^	0.078 ± 0.014^**a**^	0.075 ± 0.015^**a**^	
β-glucosidase		0.068 ± 0.004^**a**^	0.088 ± 0.011^**a**^	0.071 ± 0.010^**a**^	0.087 ± 0.006^**a**^	
NAG		0.071 ± 0.012^**a**^	0.080 ± 0.013^**a**^	0.081 ± 0.007^**a**^	0.084 ± 0.009^**a**^	
α-mannosidase		0.067 ± 0.001^**a**^	0.096 ± 0.008^**a**^	0.077 ± 0.008^**a**^	0.087 ± 0.031^**a**^	
**α-fucosidase**		**0.067 ± 0.006**^**a**^	**0.110 ± 0.018**^**b,c**^	**0.080 ± 0.006**^**a,b,c**^	**0.110 ± 0.019**^**b,c**^	

**Figure 6:**
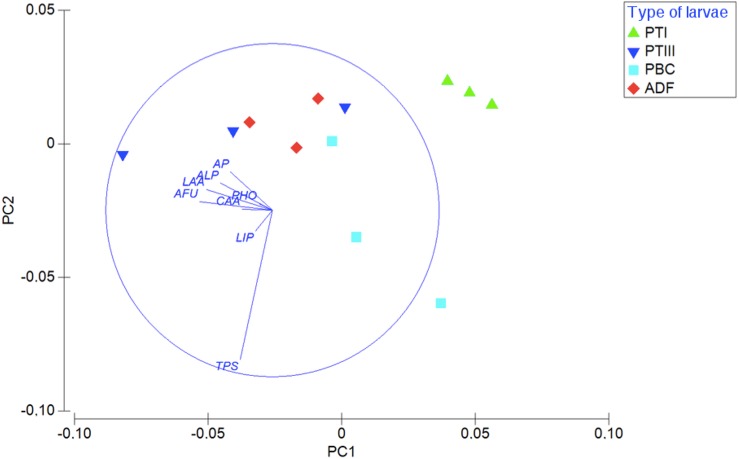
Digestive enzymatic performance of polychaete larvae. Principal component analysis (PCA) plot of enzymatic activity data of 180 mg of larval tissue dry weight of *B. wellingtonensis* planktotrophic Type I (PTI), planktotrophic Type III (PTIII) and adelphophagic (ADF) larvae, planktotrophic larva of *B. chilensis* (PBC). Variables (enzymes) are indicated on the vector plot. ALP, alkaline phosphatase; LAA, leucine arylamidase; PHO, naphthol-AS-BI phosphohydrolase; AP, acid phosphatase; LIP, lipase; CAA, cystine arylamidase; TPS, trypsine; AFU, α-fucosidase. PC1 axis explained 55.9% and PC2 explained 19.8% of the enzymatic digestive profile between larval types.

Considering the enzymatic digestive profile, the PCA shows that planktotrophic larvae Type I are separated from the other types of larvae, while planktotrophic larvae Type III were more similar to adelphophagic larvae (Fig. 6). While PC1 axis explained 55.9% of enzymatic digestive profile between larval types, PC2 explained 19.8% of this variability. The enzymes that showed the highest contribution to explain PCA pattern were mainly from the groups of peptide hydrolases (leucine arylamidase, cystine arylamidase and trypsin) and phosphoric hydrolases (alkaline phosphatase, acid phosphatase and naphthol-AS-BI phosphohydrolase); less important were glycosidases (*α* fucosidase) and ester hydrolases (lipase) (Fig. 6).

## Discussion

This study integrated aspects related to larval feeding structures and digestive capacity as potential factors in the determination of larval feeding capacity, which can help explain the evolution of the modes of development in marine invertebrates. Our results showed that larval feeding morphology does not differ between larval feeding types in the polychaete *B. wellingtonensis*. The experiments also revealed that qualitatively both adelphophagic and planktotrophic larvae of *B. wellingtonensis* are able to ingest dissolved protein. The digestive enzymatic activity per larva was higher in adelphophagic larvae in comparison to the other types of larvae. Global digestive enzymatic activity profile showed higher differences between planktotrophic larvae of Type I of *B. wellingtonensis* and *B. chilensis*, and adelphophagic larvae, than between planktotrophic Type III and adelphophagic larvae. These results suggest that both digestive enzymatic activity and enzymatic profile of larvae might be playing an important role in the evolution of feeding behaviors of marine invertebrates at early developmental stages.

In gastropods with encapsulation, another extra-embryonic food source is intracapsular fluid, which is partially composed of dissolved albumin (Chaparro, Charpentier & Collin, 2002; Brante, Fernandez & Viard, 2009; Chaparro et al., 2012). The ability of larvae to ingest and assimilate dissolved albumin inside the capsule may have a significant effect on their intracapsular development (Moran, 1999; Brante, Fernandez & Viard, 2009). The observation that planktotrophic and adelphophagic larvae of *B. wellingtonensis* may ingest fluorescent dissolved albumin suggests that this larval capacity would be observed across different taxonomic groups showing encapsulation behavior. However, we still need more work to confirm the presence and quantify the concentration of albumin in the intracapsular fluid of polychaete capsules and to evaluate the potential effect on larval development.

In *B. wellingtonensis*, as a poecilogonous species, some females produce planktotrophic Type III and adelphophagic larvae in a brood, while other females lay only planktotrophic larvae (Type I). The factors explaining why some larvae have the capacity to use nurse eggs as food are not yet clear; even more when no apparent larval morphological differences are observed, and both types of larvae may ingest dissolved protein as we showed in this work. From the 19 enzymes tested, two peptide hydrolases (leucine arylamidase and trypsin), two phosphoric hydrolases (acid phosphatase and napththol-AS-BI phosphohydrolase) and three glycosidases (*β* galactosidase, *β* glucuronidase and *α* fucosidase) showed clear differences between larval types, with adelphophagic larvae of *B. wellingtonensis* evidencing, as a general pattern, higher activity per individual than the other larval types. The exopeptidase leucine arylamidase and phosphatases participate in the metabolism of internal reserves, such as yolk reserves, in eggs and embryos of crustacean species (Saborowski et al., 2006). On the other hand, trypsin is a typical digestive endopeptidase enzyme which facilitate the digestion of alimentary proteins in the gut (Dall & Moriarty, 1983; Saborowski et al., 2006); similarly, carbohydrase enzymes allow digestion of prey (Saborowski et al., 2006). The higher activity in adelphophagic larvae of these enzymes suggests that this larval type might be using complementary nutritional mechanisms, taking advantage of internal and external energetic sources in the way of egg yolk and other eggs and larvae.

When digestive enzymatic activity profile is standardized by larval weight, planktotrophic and adelphophagic larvae in Type III reproduction evidence more similarity to each other than to Type I *B. wellingtonensis* and *B. chilensis* planktotrophic larvae. These results, in addition to the fact that adelphophagic larvae show higher digestive enzymatic activity per larvae than planktotrophic Type I, suggest that larval feeding behaviors observed in both species of *Boccardia* could be explained in part by the different capacity to digest external sources of nutrition in the way of nurse eggs and other larvae. However, it is interesting that, despite the similarity of Type III planktotrophs with adelphophages in enzyme activities (as well as structures for feeding), the Type III planktotrophs do not grow when offered nurse eggs. Oyarzun & Brante (2015) showed that when all larvae of Type III females were reared separately and fed exclusively with *Dunaliella salina*, all larvae were able to feed and developed at the same rate. In contrast, when all larvae were reared separately and fed only with nurse eggs, only a portion of them feed on those nurse eggs and continued their development (adelphophagic larvae) while others did not feed and their development remained arrested until provided with algae. The results of the present work together with the results reported by Oyarzun & Brante (2015) suggest that additional factors to digestive capacity are also influencing larval feeding type. It is possible that differences in gene expression result in adelphophages having no inhibition against cannibalism or planktotrophs not responding to conspecifics as being food. For example, although in a different reproductive type, adelphophagic embryos of the gastropod *Crepidula coquimbensis* appear to recognize kinship level and avoid full sib capsule mates as food (Brante, Fernández & Viard, 2013). In addition, Carrier, King & Coffman (2015) showed that in planktotrophic sea urchin larvae that develop completely in the water column, genes related to energetic homeostasis, cellular proliferation, growth and protein synthesis (e.g., CREB, 4E-BP, FoxO, Elk, EAAT) are overexpressed when they are cultivated in environments with high food availability. In contrast, larvae exposed to starvation down-regulate genes involved in growth and metabolic activity, while genes involved in lipid transport, environmental sensing and defense show up-regulation (Carrier, King & Coffman, 2015). Marsh & Fielman (2005) reported a more complex gene expression profile in planktotrophic than in lecithotrophic larvae of polychaetes. The authors argued that these differences might be explained by contrasting physiological functions between feeding and non-feeding larvae. Thus, although our results provide evidence to partially explain the different trophic types of larvae observed in *Boccardia* species, it is necessary to explore other complementary mechanisms that may help to understand the evolution of reproductive strategy of marine invertebrates.

In a closely related polychaete species with poecilogony, *B. proboscidea*, early development for different larval types is internally and externally similar (Gibson & Carver, 2013), which suggests that changes in early development may not account for the differences observed in larval feeding behavior and consequent development. However, same authors point out that heterochrony observed in the development of the gut and coelom in adelphophagic larvae may suggest a trade-off between rapid growth and delayed differentiation of those organs. The rapid growth by heterochrony mentioned by Gibson & Carver (2013) may be related to our results, since we showed that larger adelphophagic larvae of *B. wellingtonensis* have far more enzymatic activity than planktotrophic larvae, which may be explained by higher surface area of a larger gut for enzyme production. However, more research on the volume and expansion capability of the gut is needed.

The factors driving the evolution of different types of feeding larvae and developmental modes in polychaetes with poecilogony are unclear, as most studies only refer to general patterns of development. Evolutionary transitions and reversions between indirect (as ancestral trait) and direct development in callyptraeid gastropods have been shown to be related to the amount of food available for larval development inside protected structures (e.g., capsules and egg masses), such as yolk and nurse eggs (Collin et al., 2007). Concordantly, the retention or loss of morphological traits that allow ingestion of external sources of food, such as a mouth and larval structures used to capture particles, may play a significant role in the evolution of developmental modes in callyptraeids (Collin, 2004; Collin et al., 2007). In the present study, in addition to anatomical characteristics, we suggest that the digestive enzymatic machinery could be an important driver in the evolution of larval feeding strategies in *B. wellingtonensis*. Since the capacity of larval nutrition inside capsules is influencing developmental mode by allowing larvae to advance in development inside capsules (Chaparro et al., 1999; Collin, 2004; Oyarzun & Brante, 2014; Oyarzun & Brante, 2015), the digestive capacity of larvae to process external nutrition could explain developmental larval modes in marine invertebrates. Contrary to the low probability of evolving or re-evolving feeding structures after an evolutionary loss (Strathmann, 1978; Collin, 2004), the expression of genes associated with the production of enzymes would have a higher probability. Thus, the expression of some specific digestive enzymes may permit larvae to feed on nurse eggs within the capsule, promoting intracapsular development and reduce a free larval stage.

## Conclusions

Aspects other than morphology must be considered to study the evolution of larval feeding strategies and developmental modes in marine invertebrates, given that no apparent differences in external morphology of larvae from different feeding strategies are observed in several marine invertebrate groups*.* In the present study, we found that the activity of specific digestive enzymes in larvae of *B. wellingtonensis* is associated with different types of larval feeding strategies. Particularly, activity of enzymes associated with the digestion of internal and external sources of food showed the highest differences. We also showed that planktotrophic and adelphophagic larvae of the polychaete *B. wellingtonensis* have the potential to ingest dissolved albumin; however, the albumin content in the intracapsular fluid of this polychaete is not yet well known. These results also showed that the study of complementary mechanisms are needed to fully understand larval trophic types. Given that larval feeding strategies are closely related to how larvae develop within their capsule, factors that influence how larvae feed may play an important role in understanding the evolution and diversity of larval developmental modes in marine invertebrates.

##  Supplemental Information

10.7717/peerj.6151/supp-1 Data S1 Supplemental raw data Digestive enzymatic activity of different feeding larval. types

## Additional Information and Declarations

### Data Availability

The following information was supplied regarding data availability: The raw data of the enzymatic experiments are available as a Supplemental File.
